# Enhanced Reality Showing Long-Lasting Analgesia after Total Knee Arthroplasty: Prospective, Randomized Clinical Trial

**DOI:** 10.1038/s41598-018-20260-0

**Published:** 2018-02-05

**Authors:** Kyo-in Koo, Dae Kwon Park, Yoon Seok Youm, Sung Do Cho, Chang Ho Hwang

**Affiliations:** 1 Department of Biomedical Engineering, School of Electrical Engineering, University of Ulsan, Ulsan, Republic of Korea; 2 Department of Physical Medicine and Rehabilitation, Ulsan University Hospital, University of Ulsan College of Medicine, Ulsan, Republic of Korea; 3 Department of Orthopedic Surgery, Ulsan University Hospital, University of Ulsan College of Medicine Ulsan, Ulsan, Republic of Korea; 4 Department of Orthopedic Surgery, Dongcheon Dong-Kang Hospital, Ulsan, Republic of Korea

## Abstract

To overcome the limitation of short-term efficacy of virtual reality (VR), an enhanced reality (ER) analgesia, (combination of the VR, real-time motion capture, mirror therapy [MT]) involving a high degree of patients’ presence or embodiment was explored. Patients, who underwent unilateral total knee arthroplasty (TKA), received ER analgesia. The duration was 5 times a week, for 2 weeks for one group and 5 times a week, for 1 week in the other. Visual Analogue Scale (VAS) at rest and during movement, active knee range of motion (ROM) for flexion and extension were measured repeatedly. After screening 157 patients, 60 were included. Pre-interventional evaluation was performed at 6.7 days and ER was initiated at 12.4 days after surgery. Evaluation was performed at 5, 12, 33 days after the initiation of ER. Analgesia in the 2 week therapy group was effective until the third evaluation (*p* = 0.000), whereas in the other group, it was effective only until the second evaluation (*p* = 0.010). Improvement in ROM in the 2 week group was also maintained until the third evaluation (*p* = 0.037, *p* = 0.009). It could lay the foundations for the development of safe and long-lasting analgesic tools.

## Introduction

Analgesics are known to cause multiple adverse effects; hence, there has been a continuous research to find alternatives. However, no ideal alternative has been found yet. With the rapid development of information technology, virtual reality (VR) is emerging as an economical, safe, and convenient alternative. Its analgesic effects have been evaluated in several medical situations like burns, invasive procedures, and amputated limbs^1–4^. However, all previous publications were case reports, case series, or pilot trials, except a single on-going randomized control trial by Small *et al*.^5^. Moreover, these investigations evaluated only the short-term analgesic effect and there are no reports about the long-term effects. Since VR distraction analgesia is known to work at the level of the brain^6^, its duration is too short to be effective for long-term analgesia. If this limitation can be overcome, VR-mediated analgesia could have a wider use.

Ramachandran *et al*. first introduced analgesia using mirror therapy (MT) for phantom pain in 1996^7,8^. The proposed mechanism was that MT confounded the brain through mismatched sensory inputs from the amputated arm, inducing the brain to choose visual information instead of the amputated body sensation^9^. MT has been used for phantom pain^10^, spinal cord injury induced central pain^11^, complex regional pain syndrome^12,13^, and motor weakness after stroke^14,15^. In 2015, it was accepted that MT could induce changes in the cortical activity of the brain^16^. However, its limitation was that it was difficult to provide a perfectly synchronized mirrored image, not distorted enough to induce brain plasticity^17,18^. It is likely that VR can overcome this limitation.

Since both, VR and MT produce brain plasticity, but through different pathways, it could be postulated that a combination of VR and MT could induce synergistic or additive analgesia. A pilot trial reported the combined use of VR and analogous MT induced analgesia, in patients with rheumatic wrist arthritis^19^. The authors presumed that enhanced reality (ER: combination of VR and analogous MT using real-time image processing technique) could induce stronger brain plasticity resulting in longer-lasting effects.

## Methods

This prospective, single-blind (*i.e*. assessor-blind), parallel group, randomized (allocation ratio 1:1), single cohort clinical trial was conducted at Ulsan University Hospital from November 2013 to June 2016, registered at FDA clinical trial registry (NCT01979718 released on November 3^rd^ 2013), and it was approved and confirmed that all researches were performed in accordance with relevant guidelines and regulations by Ulsan university hospital institutional review board (UUH 2013-06-614). Patients who underwent unilateral total knee arthroplalsty (TKA) were included after providing informed consents for study participation and for inclusion of identifying images in an online open-access publication. Patients with a limited movement of the non-operated leg due to musculo-skeletal or neurological diseases, those who could not look at a monitor due to visual disturbance, could not understand visual analogue scale (VAS), and refused participation, were excluded. The recruited patients were randomly allocated to full term intervention group (FTI: intervention was provided shortly after physiotherapy for 5 weekdays over 2 weeks) or half term intervention group (HFI: intervention was provided for 1 week) by sequentially numbered containers, which were delivered to a physiotherapist in a sealed envelope, shortly before initiating the intervention. The numbers were generated using Wichmann-Hill random number algorithm. In addition to the intervention, all the patients in both groups received identical physiotherapy composing infrared radiation (wavelength 770 to 1500 nm, distance 45 cm from the patient’s body, perpendicular to the body surface) and continuous passive range of motion (ROM) exercises for 20 minutes, over 2 weeks. The patients were also encouraged to take a walk with or without an assistive device. Data of age, sex, weight, height, days from the pre-operative evaluation to surgery, educational status, Short Form Geriatric Depression Scale (SFGDS)^20^, VAS at rest and during movement, active range of movement of flexion and extension^21^, Western Ontario and McMaster Universities Osteoarthritis Index (WOMAC)^22^, graded ambulation distances^23^, 6 minute walk test^24^, and timed-stands test^25^, were collected by another physiatrist, who was blind to the allocation. Scores of all above scales, except SFGDS, and total weekly amounts of intravenous tramadol demands, adverse effects (dizziness, psychiatric distress, boredom, depressive mood, muscles twitching^26,27^, and nausea^28^) were recorded shortly after completion of each one-week session and 3 weeks after completion of the final intervention by the same physiatrist.

### Standardization of surgery and analgesia

The TKA was performed using the posterior-stabilized cruciate sacrificing method, with a medial para-patellar approach and patellar distraction. The implants were of fixed bearing type, Nexgen LPS (Zimmer, Warsaw,IN, USA), and were cemented with Simplex P (Howmedica, Rutherford, NJ), which covered the entire inner surface of the femoral implant, aligned with femoral and tibial alignment guides^29^. Standardized general anesthesia with no long-acting opioids was induced by injection of rocuronium (0.6–0.8 mg/kg), propofol (2–2.5 mg/kg), and alfentanil (15 μg/kg), maintained with 1–2% sevoflurane and 40–60% oxygen-nitrous oxide, and reversed with glycopyrrolate (7 μg/kg) and neostigmine (40 μg/kg). Alfentanil (0.25 mg) was infused intravenously if the heart rate or mean arterial pressure was more than 125% of pre-operative values. 10 mL of 1% lidocaine with epinephrine was infiltrated at the incision area for the surgical portals before the start of surgery^30^.

Based on earlier reports by Ritchie *et al*.^31^ and Hwang *et al*.^30^, analgesia was provided using celecoxib (200 mg), twice daily; acetaminophen (325 mg) and tramadol hydrochloride (37.5 mg), thrice daily; tramadol hydrocholoride (50 mg) was intravenously provided until the VAS was 40 or below. The standardized analgesia was also maintained until the patients’ first visit to the out-patient clinics.

### Enhanced reality

**Figure 1:**
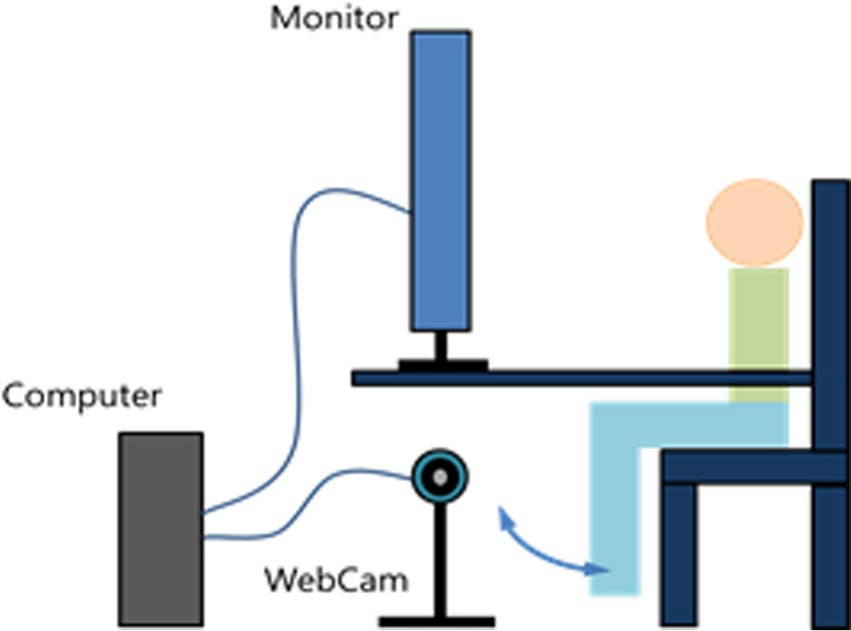
A schematic view of the enhanced reality setup. It consists of a patient positioning tool, a screening tool, an image acquisition unit, an image processing unit, and an image displaying unit. The patient positioning tool and screening tool used a conventional table and chair. A conventional WebCam, a conventional computer, a lab-made image processing program, and a conventional monitor functioned as the image acquisition tool, image processing unit, and image displaying unit.

**Figure 2:**
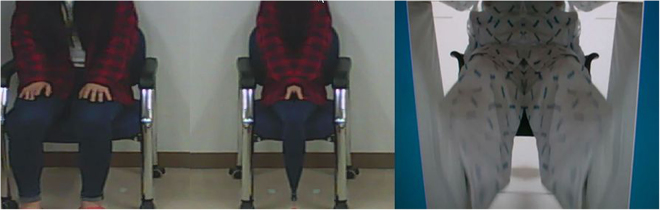
The flipped image by the processing unit of enhanced reality. The processing unit flips half of the acquired image, and copies it to the half of the acquired image. The left figure and center figure show a tested image before and after flipping. The right figure shows a patient’s flipped leg movement image during one’s intervention.

The patients were seated in a chair, thighs and trunk secured with Velcro straps, and both hands were placed on a table with spine erect, hips and knees flexed to 90 degree, and soles at 10 cm above the floor. A camera was located below the table and in front of the patient, and the distance from the patients was adjusted to allow reconstruction of virtual limbs identical to both legs, thighs, and their origin from the pelvis (Fig. 1). The intervention consisted of two sequences. The first was acquisition of a real-time embodiment and the second was of virtual limbs presence. The patients were requested to repeat voluntary free ROM movement of knee flexion and extension for 5 minutes without limitation of counts, looking at a screen showing the real-time images of their legs. Validation of embodiment of the real-time image was evaluated in random sequences^32^ (Appendix). After 5 minute breaks, the same session was repeated, but was limited to only 50 repetitions of flexion and extension, looking at the virtual limbs (Fig. 2). Validation of the virtual limbs presence was checked in random sequences again. A detailed description about ER can be found in Appendix.

### Statistical analyses

Based on an earlier report by Perera *et al*.^33^, authors defined a distance of 50 meters in 6 minute walk test, as the minimum mean difference of significance, and calculated standardized difference (0.71) using standard deviation (70) based on an earlier report by Boardman *et al*.^34^. Authors assumed power at 80%, α 95%, and calculated that the number of recruited patients required is 25 per group using Altman’s nomogram^35^, added five more patients (20% of likely dropout ratio), and finally planned to assign 30 patients to each group. Statistical Package for the Social Sciences 21.0 KO for Windows was used for the bivariate analyses. Two-sample student’s t-test, after transformation into the log in case of no normal distribution, was used to analyze the continuous variables. Wilcoxon-Mann-Whitney U test was used for the ordinal variables, repeated measures analysis of variance (ANOVA) and *post hoc* analysis was used to analyze the differences in time within the group and between groups.

## Results

**Figure 3:**
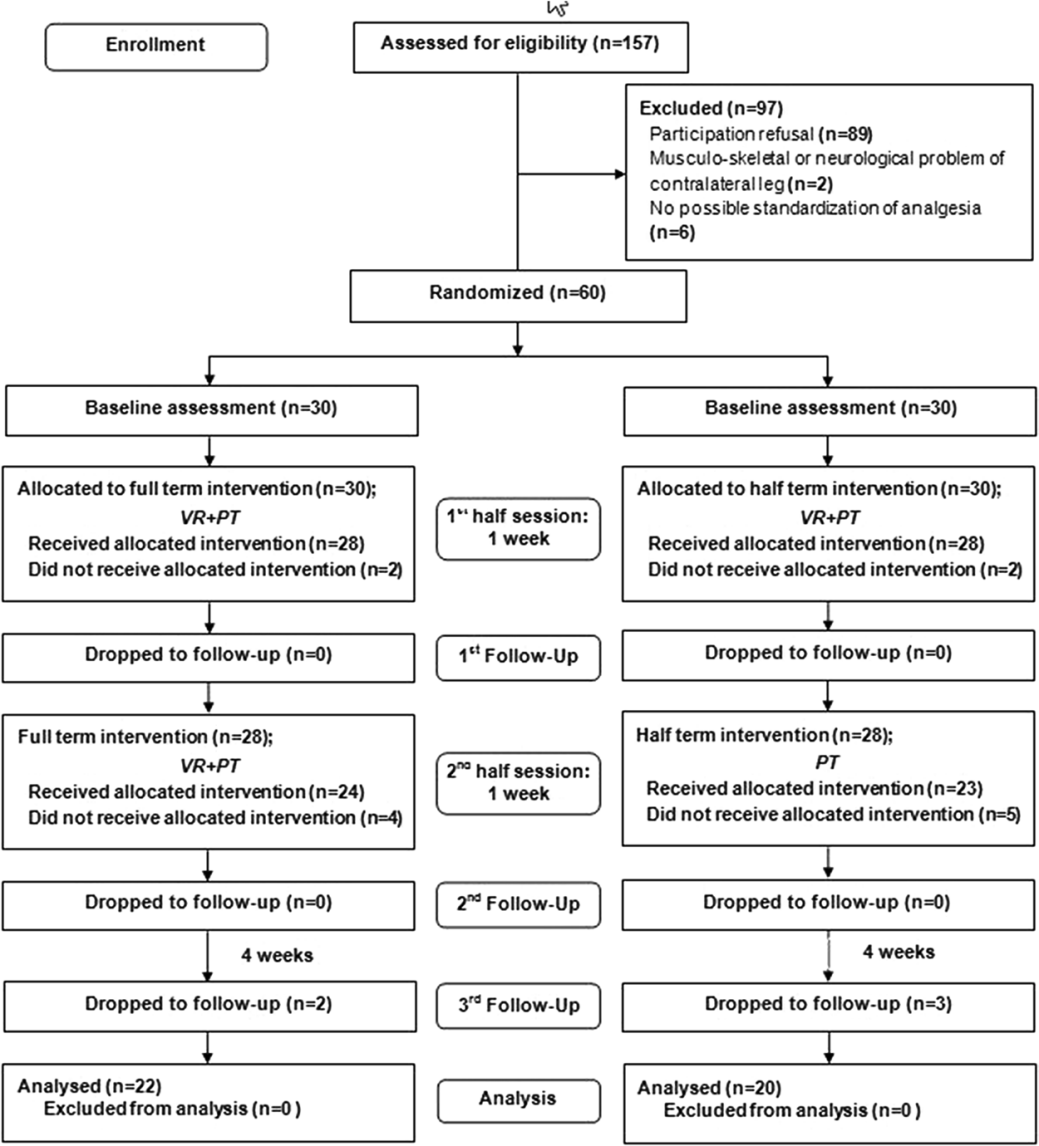
The flow diagram.

One hundred fifty seven patients who underwent unilateral TKA were assessed for eligibility, and 97 were excluded. Thirty patients were allocated to each group. Four patients were dropped during the first session (demand for analgesics: 3, toxic hepatitis: 1), nine during the second session (toxic hepatitis: 2, early discharge: 7), and five during the third session (no visit in out-patient clinic). Twenty-two in the FTI group and 20 in the HTI group were analyzed finally (Fig. 3). No significant difference in demographic characteristics was found (Table 1).

**Table 1:** Demographic characteristics.

		half term intervention group (20 patients)	full term intervention group (22 patients)	*p*-value
Age (year)		63.71 ± 5.09	65.00 ± 6.97	0.665
Sex	Female	15	17	0.608
	Male	5	5	
Weight (kilogram)		60.43 ± 12.33	63.29 ± 9.25	0.537
Height (cm)		160.57 ± 10.58	153.65 ± 7.49	0.151
Education	Elementary	8	10	0.310
	Middle school	7	8	
	High school	5	4	
Geriatric depression scale (0–15)		1.43 ± 3.78	1.47 ± 3.15	0.978
Day from surgery to preinterventional evaluation		6.71 ± 1.89	7.06 ± 2.56	0.721
Days from surgery to the first intervention		12.00 ± 1.92	12.94 ± 2.84	0.432
Days from surgery to visiting out-patient clinic		47.29 ± 8.50	45.29 ± 4.97	0.162

### Validation of real-time embodiment and virtual limb presence

In the first session, real-time embodiment of the image scored 4.0, virtual reality presence scored 4.0, and the sham stimulation −4.5. In the second session, the real-time embodiment was 4.1, virtual reality presence 3.9, the sham stimulation −4.2 (Table 2).

**Table 2:** Validation of enhanced reality.

	half term intervention group	full term intervention group	*p*-value
Real-time embodiment (the first week) (−5 to 5)	3.91 ± 0.56	4.01 ± 0.64	0.349
Virtual limb presence (the first week) (−5 to 5)	3.84 ± 0.66	4.12 ± 0.51	0.272
Sham stimulation (the first week) (−5 to 5)	−4.74 ± 0.34	−4.35 ± 0.32	0.416
Real time embodiment (the second week)	—	4.06 ± 0.51	—
Virtual reliability (the second week)	—	3.86 ± 0.70	—
Sham stimulation (the second week) (−5 to 5)	—	−4.19 ± 0.41	—

### Functional scales and painkiller usage

No significant difference was found between the pre-interventional evaluation and the evaluation at the three time points after intervention (Table 3). The routine provision with the standardized analgesia controlled the patients’ pain so enough during the second weeks of the interventions that no intravenous injections of tramadol hydrocholoride happened. Change in WOMAC scale showed no significant differences in time within the group and between groups (*p* = 0.081, F: 11.53 and *p* = 0.078, F: 9.61). All the patients showed 4 grades in the graded ambulation distance.

**Table 3:** Comparison of the functional scales and the tramadol usage at each evaluation time-point.

		half term intervention group	full term intervention group	p-value
Pre-interventional evaluation	WOMAC (0–96)	40.86 ± 10.89	37.47 ± 12.71	0.544
	6 minute walk test (meter)	126.86 ± 64.05	146.59 ± 89.24	0.602
	Timed-stands test (second)	24.71 ± 5.25	26.8 8 ± 7.11	0.534
The first week	Number of tramadol weekly consumption	3.00 ± 2.24	1.98 ± 1.53	0.288
	WOMAC	35.00 ± 15.01	30.41 ± 11.57	0.426
	6 minute walk test	282.57 ± 132.19	220.77 ± 91.19	0.199
	Timed-stands test	22.43 ± 5.86	22.57 ± 3.31	0.418
The second week	Number of tramadol weekly consumption	2.14 ± 2.41	1.33 ± 1.70	0.534
	WOMAC	19.14 ± 13.98	21.12 ± 9.74	0.695
	6 minute walk test	337.71 ± 85.77	290.64 ± 80.26	0.213
	Timed-stands test	19.71 ± 5.29	20.44 ± 2.96	0.400
The fifth week	WOMAC	10.86 ± 10.84	14.59 ± 9.14	0.398
	6 minute walk test	407.00 ± 83.62	353.35 ± 82.35	0.163
	Timed-stand test	19.00 ± 6.16	19.29 ± 2.80	0.867

### Difference in duration of analgesia and ROM

**Figure 4:**
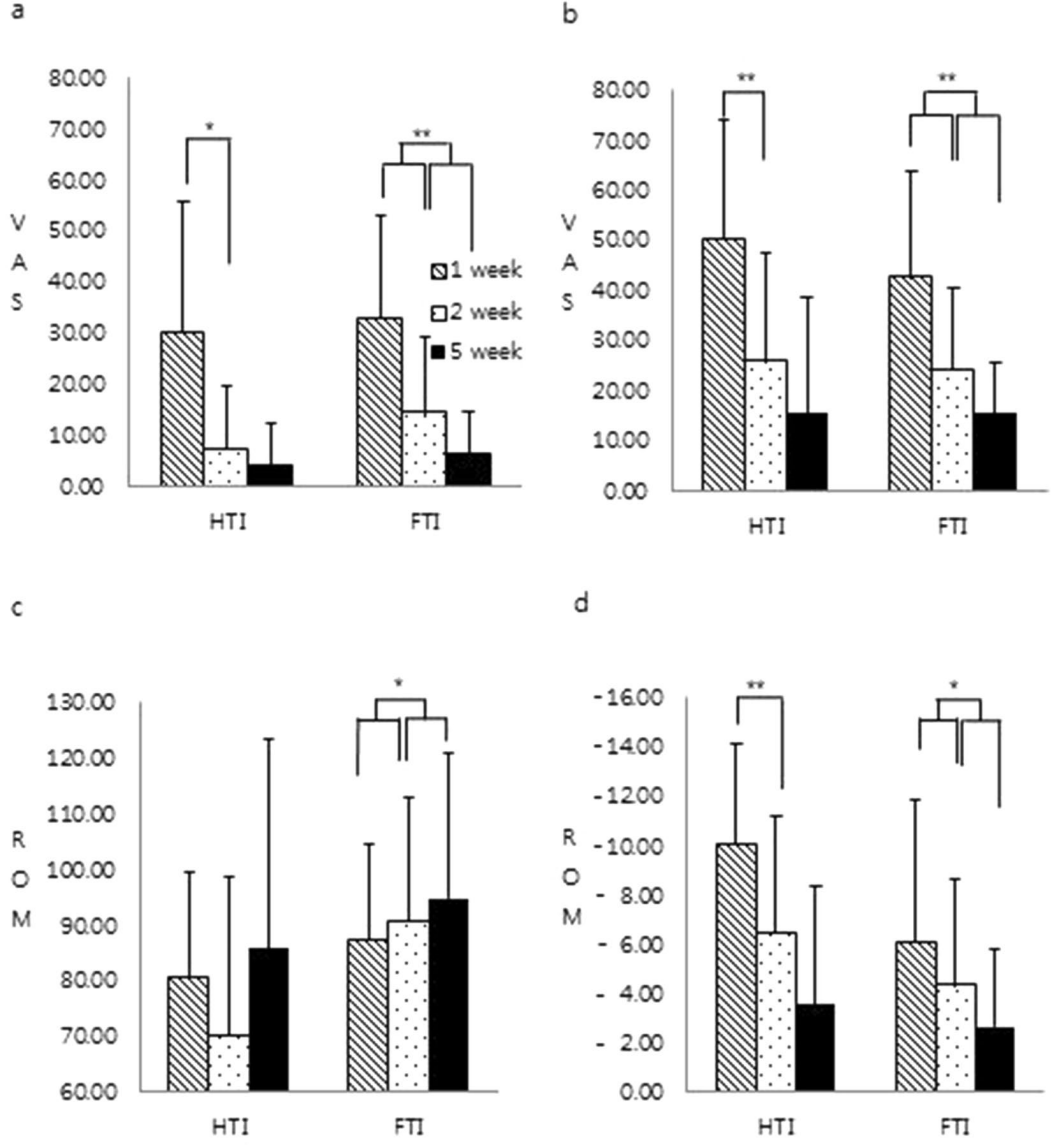
Difference in duration of analgesia and ROM. (**a)** and (**b)** Continuous significant improvement of the VAS at rest and movement was seen over 5 weeks in the FTI group and improvement in the HTI was limited to 2 weeks. (**c)** Only the FTI group showed continuous significant improvement of active knee flexion over 5 weeks. (**d)** Continuous significant improvement of active knee extension was seen over 5 weeks in the FTI and improvement in the HTI was limited to 2 weeks. FTI: full term intervention group, HTI; half term intervention group. *p < 0.05, **p < 0.01.

The FTI group showed a continuous and significant change in VAS at rest over 5 weeks (*p* = 0.000, F: 23.65). However, the HTI group showed significant change in VAS only between the first and second week (*p* = 0.010, F: 6.90) (Fig. 4a). The VAS on movement showed similar results: significant improvement in the FTI group over 5 weeks (*p* = 0.000, F: 28.39) and in the HTI group improvement was seen only between the first and second week (*p* = 0.008, F: 7.43) (Fig. 4b). No significance was found in VAS analyses between the groups.

A significant difference in time within the group was demonstrated in active ROM of knee flexion and extension in the FTI group (*p* = 0.037, F = 4.91 and *p* = 0.009, F = 7.18), but group-by-time interaction in ROMs was not noticed. The *post hoc* analysis revealed that a continuous and significant improvement in active ROM of flexion and extension was found over 5 weeks in the FTI group, but significant improvement in active ROM of extension was found only between the first and second week in the HTI group (Fig. 4d).

## Discussion

Improvement in VAS at rest or during movement was noticed in both groups. This is similar to previous reports of VR^1–4^. However all previous reports were case-series or crossover trials, and evaluated only the immediate analgesia during ROM exercises, burn wound debridement, or chemotherapy. Two systemic reviews concluded that VR has the potential to be an useful analgesic tool, but more evidence is required^36,37^. With no randomized controlled trials except a single on-going trial^5^, it is important to check the long-lasting effect. In comparison within the group, the FTI group showed a significant improvement which was maintained at 3 weeks after discontinuation of the intervention; however, the improvement in HTI group lasted only one week. Considering the duration of the intervention in the two groups (HTI: one week vs. FTI: two weeks), this indicates that long-lasting analgesia is dose-dependent. A few trials evaluated the effectiveness of analgesia with multiple VR sessions^38,39^. Hoffman *et al*. reported there was no decrease in pain until 7 sessions, in 7 burn patients performing ROM exercises^38^. Faber *et al*. found that analgesia was maintained significantly until the third day, but not beyond, in 36 patients undergoing burn dressing^39^. However, their evaluation period was too short following the VR to be comparable with the current results.

The current long-lasting effect can be explained through the pathway of induction of brain plasticity in brief. The VR induces analgesia through distraction phenomena acting on the pain matrix (the anterior cingulate cortex, primary and secondary somatosensory cortex, insula, thalamus)^6,40,41^. It is known that neuroplasticity can be induced in response to stimuli in a matured adult brain^42,43^ and pain can be one of these stimuli^44,45^. Similarly the MT induces change in cortical activity of the human brain^16^, and it has been used in various medical fields^10–15^. Visual input dominates other somatosensory efferent signals for proprioceptive perception of the brain^46^. MT confounds the brain^32^, which recognized the reflected visual feedback as a well-functioning limb image, and induces neuro-plasticity of the brain in charge of the contralateral body^47–49^. Considering that the motor cortex activation provides analgesia^50^ and that the motor cortex gets activated even by looking at the movement of another person’s extremity^51^, in the current trial it might have induced synergistic analgesia with VR distraction, by watching the image of a well-functioning operated leg. Similar to the current trial, VR using the concept of MT has been attempted earlier. Desmond *et al*. reported that immediate analgesia was noticed in 1 of 3 patients due to VR, in which the virtual forearm controlled by the contralateral hand, was superimposed on the amputated arm^50^. Cole *et al*. reported analgesia in 10 amputations among 14 leg or arm amputations using a similar design^52^. Only one trial, reported long-lasting analgesia of 1 week between sessions, in a protocol of 5 sessions, once a week. The VR mirror visual feedback induces analgesia lasted for 1 week in 4 out of 5 patients with complex regional pain syndrome^53^. However, imaginary movements alone could exacerbate pain in spinal cord injury^54^ or complex regional pain syndrome^55^. The above trials used augmented virtual limbs, rather than real limbs like the MT; however, this could lead to poor embodiment. Considering that high degree of embodiment in the MT^56,57^ and the sense of presence in the VR distraction^37,53,58^ are essential, it is critical that the stimuli should be strong enough to evoke the brain plasticity^52^ and the validity of embodiment and the sense of presence might be the determining factors for better results.

Most trials on VR distraction analgesia used a single self-reporting question for validity of the presence. Six patients reported the strongest feeling of going-inside the VR among 11 patients undergoing the VR during burn wound debridement in a trial by Hoffman *et al*.^2^ and 8 felt virtual sensation among 14 amputated patients^53^. The mean value of validity of presence was 56 (0 to 100 rating) during the VR in 7 burn patients who took ROM exercises^38^. Moreover, all earlier reports were preliminary. Although a questionnaire with multiples parameters used by Witmer *et al*. showed higher reliability^59^, it was used only in a single clinical trial of 8 pediatric burn patients in which the mean score was 4.8 (0 to 7 rating)^60^. However, authors used a questionnaires consisting of six items, to check for real-time embodiment, virtual limb presence, and sham stimuli (−5 [not at all] to 5 [perfect] rating)^32^. In the first session, the real-time embodiment scored 4.0, the virtual limb 4.0, and the sham stimulation −4.5, and in the second session, the real-time embodiment was 4.1, the virtual limb 3.9, the sham stimulation −4.2. These scores were much higher than scores of the previous trials, indicating that ER might induce long-lasting analgesia through more potentiated brain plasticity.

Comparing the ROM improvement within the group, similar results were noticed: The FTI group showed significant improvement until 3 weeks after the discontinuation of the intervention, whereas the improvement in the HTI group was only for one week after. Sarig-Bahat *et al*. reported that a single session of VR, in which each patient was encouraged to catch up flies with spray, increased cervical ROM (generally 10 degree or more) in 67 patients with/without neck pain^61^. This is in accordance with the current results, however, only the immediate effect was evaluated. Considering that the same VR showed an error range in ROM assessment of up to 7.2° in flexion and extension and to 16.1° in rotation^62^, the current trial may be the first evidence that the VR improves active ROM.

No major or minor adverse event was noticed although simulator sickness in the VR^28,38^ and dizziness, emotional discomfort, depressive mood, and muscle twitching in the MT^26,27^ were reported. However, objective evaluation, similar to a study by Keshavarz and Hecht^63^, was not performed.

The current study had several limitations. Per-protocol analysis was used instead of intention-to-treat analysis, because the protocol was violated in 18 patients. The effect size could be overvalued by per-protocol analysis^64^ and the large number of dropouts (18 of 60), which is a high number for a study of this size. Majority (76%) of the patients in the current trial were females. Considering the difference in visual perception between sexes^65–67^, no sub-group analyses regarding sex may be warranted. Although a significant difference in time within the group over 5 weeks in VAS and active ROM of the knee was approved in the current trial, it could not gain generalizability because its action induced no significant improvement of the functional scales and was restricted to the knee, which the intervention was targeted towards. It could be because the period of intervention was not long enough; hence, a clinical trial is necessary to determine the therapeutic time window and dosage. Another limitation was, not checking the degree of neuroplasticity using electro-diagnostic or imaging evaluation.

## Electronic supplementary material

Appendix 1& 2
