# Supplementary material for: Enhanced Reality Showing Long-Lasting Analgesia after Total Knee Arthroplasty: Prospective, Randomized Clinical Trial
Source: Sci Rep. 2018 Feb 5;8:2343. doi: 10.1038/s41598-018-20260-0 (PMC5799299; doi:10.1038/s41598-018-20260-0)
Supplement: Supplementary file 1 — Appendix 1& 2 [file 41598_2018_20260_MOESM1_ESM.pdf]

Enhanced Reality Showing Long-Lasting Analgesia after Total Knee Arthroplasty: Prospective, Randomized Clinical Trial

Kyo-in Koo, Ph.D., Dae Kwon Park, M.D., Yoon Seok Youm, M.D., Ph.D., Sung Do Cho, M.D., Ph.D., Chang Ho Hwang, M.D., Ph.D.

Appendix 1. Validation of the real-time image embodiment and the virtual limbs presence

Validation of real-time embodiment

1. Do you feel the legs in the screen as your legs?

-5 (never), -4, -3, -2, -1, 0, 1, 2, 3, 4, 5 (definitely)

2. Do you feel the legs in motion in the screen as your legs?

-5 (never), -4, -3, -2, -1, 0, 1, 2, 3, 4, 5 (definitely)

3. Do you feel your legs warm?

-5 (never), -4, -3, -2, -1, 0, 1, 2, 3, 4, 5 (definitely)

Validation of virtual limb presence

1. Do you feel the legs in the screen as your legs?

-5 (never), -4, -3, -2, -1, 0, 1, 2, 3, 4, 5 (definitely)

2. Do you feel the legs in motion in the screen as your legs?

-5 (never), -4, -3, -2, -1, 0, 1, 2, 3, 4, 5 (definitely)

3. Do you feel your legs warm?

-5 (never), -4, -3, -2, -1, 0, 1, 2, 3, 4, 5 (definitely)

Appendix 2. A detailed description about cocktail of virtual limbs, motion capture, and mirror therapy

Our cocktail system consists of a patient positioning tool, a screening tool, an image acquisition unit, an image processing unit, and an image displaying unit. The image acquisition unit provides patients' leg movement to an image processing unit in real-time. Any Windows-compatible WebCam device can be used as the image acquisition tool. A C920r of Logitech, U.S.A. was used as the image acquisition tool. The image processing unit is a lab-made image processing program operating on a conventional Windows-based computer. The image processing program was developed by authors using Open Source Computer Vision (OpenCV), a library of programing functions for real-time computer vision, and Microsoft Foundation Class (MFC), a library of programing functions for windows control in the Microsoft Windows Operating System, based on Visual Studio (Microsoft, U.S.A.), an integrated program development environment. At the first section of the intervention, the image processing unit provides patients' leg movement without any image processing to the image displaying unit, a conventional 1920 by 1080 resolution computer monitor. Through the displaying unit, the patients watch their real leg movement in real-time for embodiment augment of the displayed images. At the second section, a virtual image is processed and transferred to the displaying unit by the processing unit. Processing time is short enough that the patients cannot feel any movement delay in the virtual movement images. The processing unit replaces the operated-leg movement by the not-operated-leg movement. More specifically, half of the acquired image (the not-operated-leg part) is flipped and copied to another half of the acquired image (the operated-leg part). Eventually, the patients watch the not-operated-leg movements only at the second section, instead of their operated-leg and not-operated-leg movement.
